# Pilomatrix Carcinoma of the Antecubitum

**DOI:** 10.7759/cureus.6821

**Published:** 2020-01-30

**Authors:** Michael D Eckhoff, Joshua Tadlock, Lisa A Kafchinski

**Affiliations:** 1 Orthopedics, Texas Tech University Health Sciences Center El Paso, Paul L. Foster School of Medicine, El Paso, USA; 2 Orthopedics, Texas Tech University Health Sciences Center El Paso, El Paso, USA

**Keywords:** pilomatrix carcinoma, tumor surgery, orthopedic oncology

## Abstract

We present the case of a 46-year-old male with atypically large left elbow pilomatrix carcinoma present for 10 years with emergent excision after developing life-threatening hemorrhage. Pilomatrix carcinoma is a dermal-based malignant tumor typically of the head and neck region. Histopathology shows islands of basaloid cells, shadow cells, and fibromyxoid fibroma. Reoccurrence is seen in 23% of cases on an average of six months after primary excision. The current standard of care is wide excision with close follow-up.

## Introduction

Pilomatrix carcinoma is a rare, low-grade, malignant skin tumor of the hair matrix initially described in 1980 with approximately 125 cases reported in the literature [1-4]. It is related to pilomatrixoma, a common benign skin tumor. Pilomatrix carcinoma is characterized by a slow growth, firm, mobile nodule often misdiagnosed on initial evaluation [2,5]. They typically occur in middle-aged white males in the head and neck region, although there are several reported cases arising in extremities [3,4,6-9]. When caught early, these lesions are often treated by a dermatologist with Mohs surgery [10-12].

Histologically, these demonstrate poorly circumscribed tumors with basaloid and “ghost” cells and frequent metastases [1,13]. The immunohistopathologic analysis demonstrates mutations in CTNNB1, a β-catenin gene [14,15]. Rarely these tumors will show sarcomatoid features as well [9]. The recurrence rate is high with 23% after wide excision, and with metastasis occurring in 13% [3,16].

Our case demonstrates a patient with an exophytic mass to the upper extremity with uncontrolled hemorrhage and local reoccurrence within four months of initial excision.

## Case presentation

**Figure 1:**
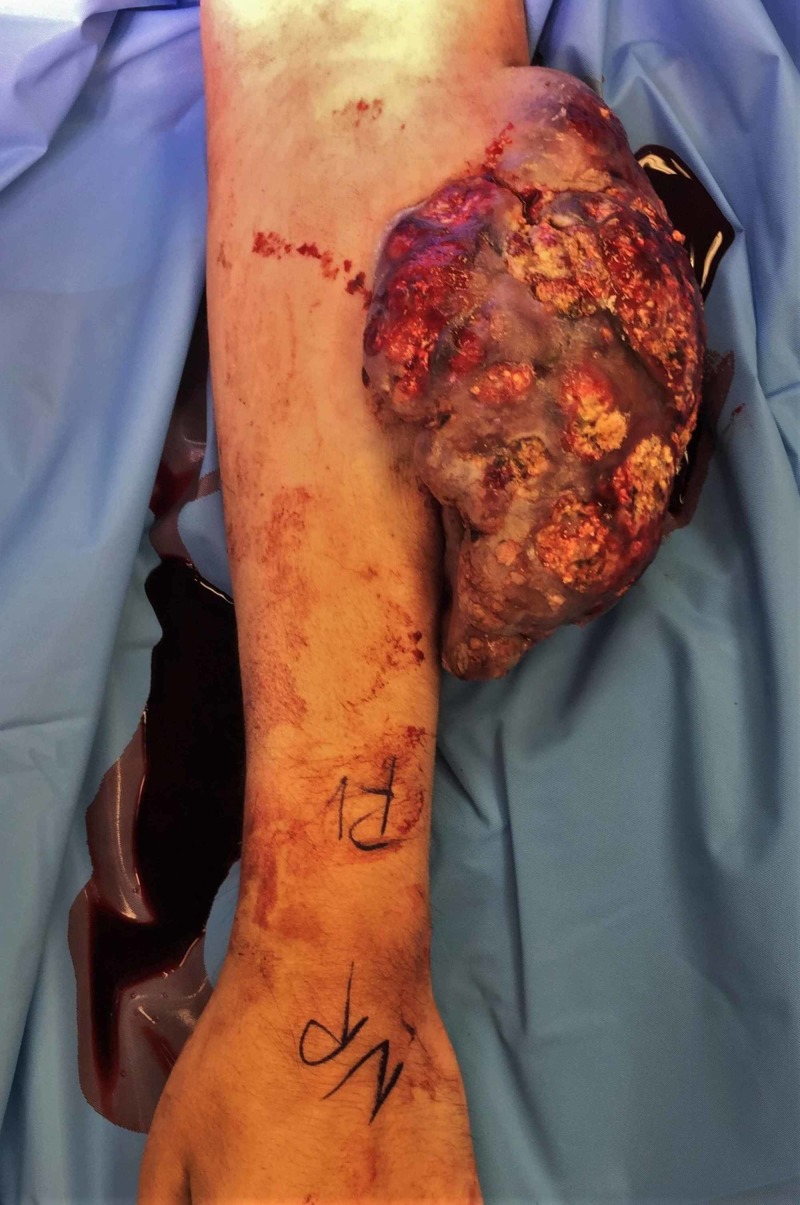
Preoperative image Preop image of patient's arm with exophyic mass over antebrachium

**Figure 2:**
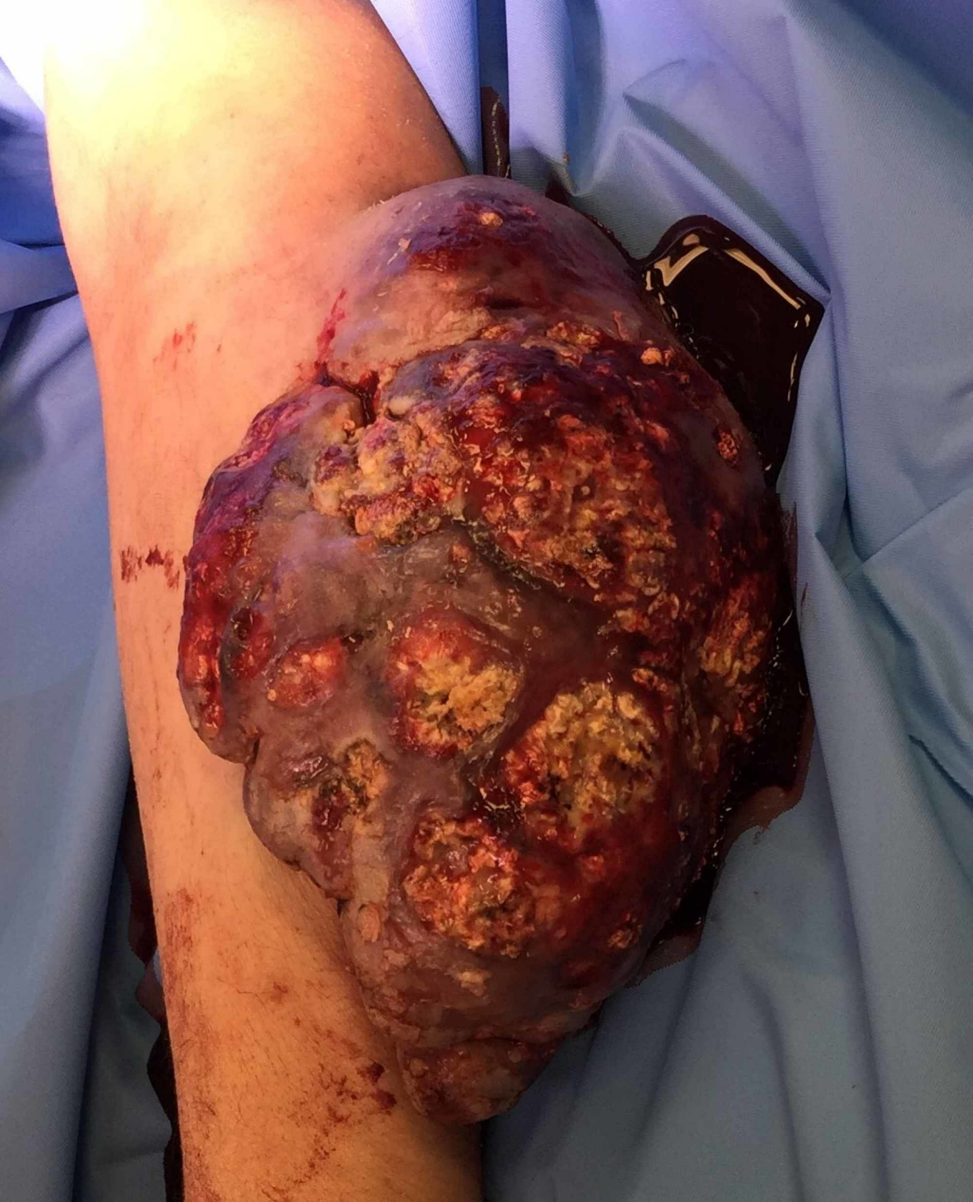
Close up of exophytic mass Closer image of preoperative patient's arm with exophytic mass

A 46-year-old male presented with a greater than 10-year history of a left arm wound. He initially developed a wound on his left antecubital region after a spider bite. The wound that developed nearly healed before becoming increasingly friable, vascularized, and enlarging. Starting about 1 cm in diameter it progressed to 15x12 cm in size with the greatest growth in six months prior to excision. On evaluation by the senior author, it was foul smelling, painful, draining fluid, and bled easily. His preoperative MRIs are shown in Videos 1, 2. The patient was scheduled for surgery 10 days later. Five days later, however, the patient presented to the emergency department with uncontrolled hemorrhage from the lesion, necessitating tourniquet application, multiunit transfusions, and emergent surgical excision. Figures 1, 2 show the images obtained immediately prior to excision.

**Video 1:** Axial T2 with contrast Preoperative axial T2 MRI with contrast demonstrating heterogeneous components with septations

**Video 2:** Sagittal T2 MRI preoperatively Preoperative T2 MRI of arm demonstrating exophytic mass with T2 enhancement of heterogeneous components

After the index excision, the patient required additional irrigation and debridement on postoperative day (POD) 3. His skin defect was left uncovered pending final pathology. The patient returned for limited tumor bed re-excision to obtain 5 mm margins and placement of biologic dressing in anticipation of split-thickness skin grafting (STSG) on POD 12. Negative margins were obtained with a re-excision surgery. He subsequently underwent STSG, which he healed without incident.

Pathologic review of the initial excised mass demonstrated extensive areas of necrosis and hemorrhage with focal areas of myxoid and cystic degeneration. Microscopic examination was remarkable for an increased mitotic index, and desmoplastic and lymphovascular invasion. These findings are consistent with pilomatrix carcinoma.

**Figure 3:**
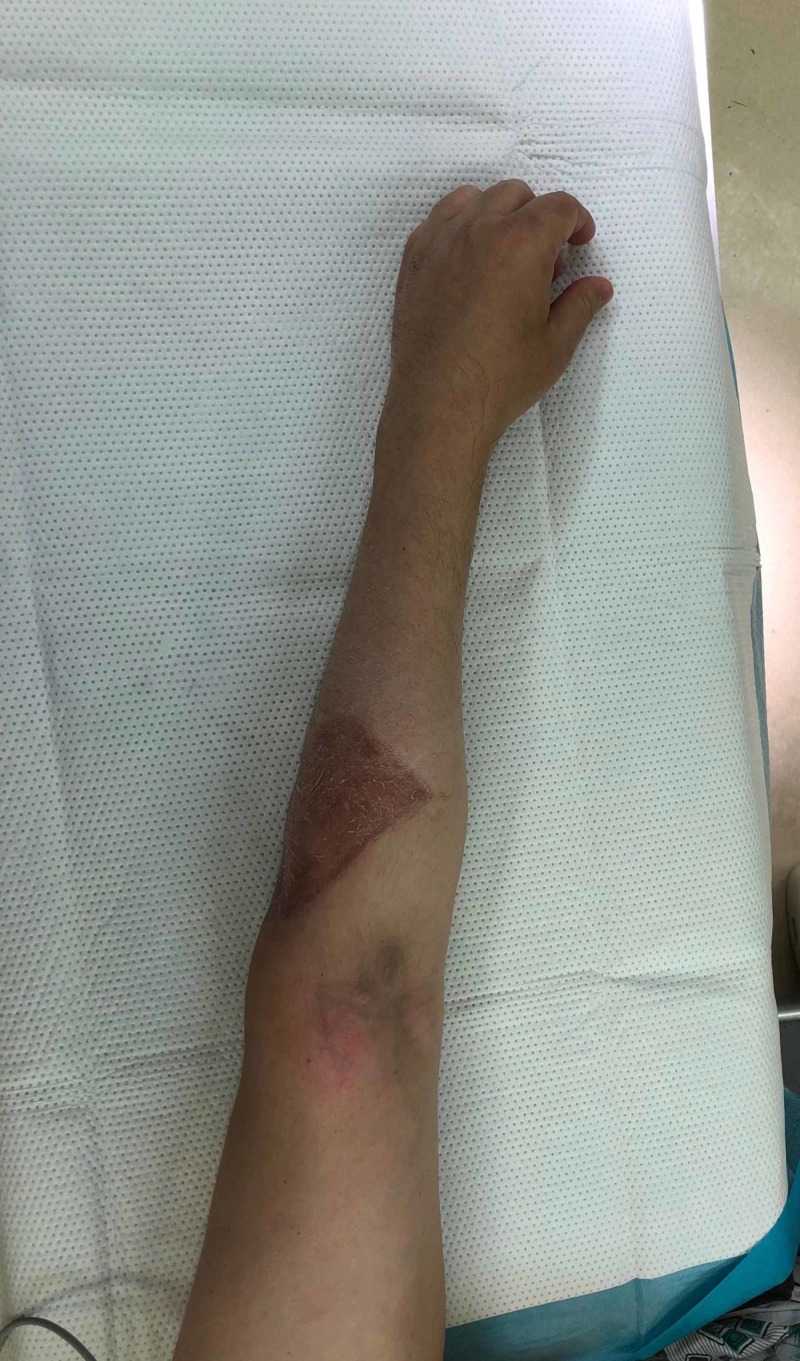
Preop image before local reoccurrence removal

Seven months after resection, the patient presented with a three-month history of left antecubital mass that was 2x4 cm and growing. MRI and positron emission tomography (PET) scan were obtained demonstrating left antecubital lesion with the involvement of cephalic vein and avid on PET scan. These are shown in Videos 3-5. A clinical photo of his arm is shown in Figure 3 after healing his skin graft from the initial surgery, but before excision of the reoccurrence. No distant metastases were identified. One month later, the patient underwent surgical excision of the new lesion with primary closure. Pathologic review at this time was also consistent with pilomatrix carcinoma. The patient healed from this procedure without incident. At the time of publication, the patient was 24 months out from index procedure without evidence of new reoccurrence or metastasis.

**Video 3:** Sagittal T2 MRI of local reoccurrence Sagittal T2 weighted MRI of local reoccurrence demonstrating similar appearance as the original tumor

**Video 4:** Axial T2 MRI of local reoccurrence Axial T2 weighted MRI of local reoccurrence demonstrating similar appearance as initial tumor

**Video 5:** PET/CT of reoccurrence PET/CT scan of the patient after reoccurrence demonstrating increased uptake in the left arm. The video shows from the level of the stomach to the bladder. On the right side of the video (patient's left arm) you can see an area of increased uptake from the local reoccurrence of pilomatrix carcinoma. PET, positron emission tomography

## Discussion

Pilomatrix carcinoma is a skin-based malignant tumor often appearing on the head and neck region in adult males. These tumors are exceptionally rare. This paper discusses an interesting case with a presentation in the antecubitum requiring emergent surgical excision and subacute local reoccurrence.

Our patient’s tumor had characteristics consistent with what has been previously described. However, the tumor was quite large, measuring 12x15 cm, where the average in a study by Herrmann et al. was 3.8 cm in diameter, although there were samples up to 20 cm in size [3]. The percentage of these tumors to reoccur locally is 23% and as metastatic lesions in 13% of patients. The average time to reoccurrence has been found to be six months, even when appropriate wide excision of 2-3 cm is performed. This time frame was consistent with our case. Fortunately, our patient has not shown evidence of metastasis at 24-month follow-up.

Additionally, these tumors are highly vascular; however, vascularity does not guide the treatment course for these tumors [17]. In most described cases, the tumors are friable and bleed easily. Our patient's tumor had significant enough vasculature that life-threatening hemorrhage occurred requiring tourniquet application, transfusion, and emergent surgical excision to control.

The pathologic review of the tumor demonstrated extensive necrosis with focal areas of myxoid stroma, cystic degeneration, high mitotic index, and invasion into desmoplastic and lymphovascular structures. This is consistent with previous studies demonstrating islands of basaloid cells, areas of necrosis, shadow cells, and fibromyxoid stroma. Pilomatrix carcinoma is differentiated from a pilomatrixoma by the amount of nuclear pleomorphism, mitotic atypia, and local invasion of neural and vascular elements [2,8,10,13].

Current standard of care is wide resection with margins of 5-30 mm [11,13,14]. When the lesion is identified early, there is good evidence for successful treatment with Mohs surgery as the average size is 3.8 cm in diameter [10-12]. After treatment, these patients need close follow-up due to the relatively high risk of local reoccurrence and metastasis even with wide excision [3]. The future of care in these lesions will focus on investigating adjuvant and neoadjuvant modalities to include radiotherapy, electron-beam radiation, and chemotherapy to decrease the chance for reoccurrence [18,19].

## Conclusions

We presented the case of a 46-year-old male who developed an antecubital pilomatrix carcinoma, a dermal-based malignancy that is characterized by islands of basaloid cells and shadow cells. The case presented is unusual with atypically large tumor size, emergent treatment due to uncontrolled hemorrhage, and local recurrence. The patient was initially treated with emergent resection and staged tumor bed re-excision for the original tumor. He subsequently underwent excision of the local recurrence as well. Local recurrence is common after wide resection, as demonstrated in this case.
